# Novel Information on the Epitope of an Inverse Agonist Monoclonal Antibody Provides Insight into the Structure of the TSH Receptor

**DOI:** 10.1371/journal.pone.0031973

**Published:** 2012-02-16

**Authors:** Chun-Rong Chen, Larry M. Salazar, Sandra M. McLachlan, Basil Rapoport

**Affiliations:** 1 Thyroid Autoimmune Disease Unit, Cedars-Sinai Medical Center, Los Angeles, California, United States of America; 2 University of California Los Angeles School of Medicine, Los Angeles, California, United States of America; Cardiff University, United Kingdom

## Abstract

The TSH receptor (TSHR) comprises an extracellular leucine-rich domain (LRD) linked by a hinge region to the transmembrane domain (TMD). Insight into the orientation of these components to each other is required for understanding how ligands activate the receptor. We previously identified residue E251 at the LRD-hinge junction as contributing to coupling TSH binding with receptor activation. However, a single residue cannot stabilize the LRD-hinge unit. Therefore, based on the LRD crystal structure we selected for study four other potential LRD-hinge interface charged residues. Alanine substitutions of individual residues K244, E247, K250 and R255 (as well as previously known E251A) did not affect TSH binding or function. However, the cumulative mutation of these residues in varying permutations, primarily K250A and R255A when associated with E251A, partially uncoupled TSH binding and function. These data suggest that these three residues, spatially very close to each other at the LRD base, interact with the hinge region. Unexpectedly and most important, monoclonal antibody CS-17, a TSHR inverse agonist whose epitope straddles the LRD-hinge, was found to interact with residues K244 and E247 at the base of the convex LRD surface. These observations, together with the functional data, exclude residues K244 and E247 from the TSHR LRD-hinge interface. Further, for CS-17 accessibility to K244 and E247, the concave surface of the TSHR LRD must be tilted forwards towards the hinge region and plasma membrane. Overall, these data provide insight into the mechanism by which ligands either activate the TSHR or suppress its constitutive activity.

## Introduction

The glycoprotein hormone receptor (GPHR) structure consists of three distinct components. Like all members of the G protein coupled receptor (GPCR) family, a serpentine membrane spanning domain is responsible for communicating with the intracellular signaling mechanism. Based on the solved crystal structure of this component in other rhodopsin-like GPCR family members [1]–[4], molecular modeling of the GPHR transmembrane domain (TMD) provides a reasonable structural representation. The second GPHR domain, entirely extracellular and comprising the major ligand binding site, consists of leucine-rich repeats (approximately 240 amino acid residues after removal of the signal peptide). The structure of this leucine-rich repeat domain (LRD) is even more clearly established than that of the TMD, with crystal structures available for both the FSH- [5] and TSH- [6] receptors. The structure of the third GPHR component, a hinge region linking the LRD to the TMD (approximately 100–150 amino acid residues in different family members), is unknown. Insufficient homology to other known proteins precludes reliable molecular modeling. At least in the case of the TSHR, the hinge region contains a portion of the ligand binding site [7]–[10].

Without insight into the relative orientation to one another of the GPHR components (LRD, hinge and TMD) it is not possible to understand, even from ligand-LRD crystal structures [5], [6], the mechanism by which ligand binding triggers intracellular signaling. All three GPHR components have not been crystallized as a unitary structure. Consequently, GPHR models have varied widely. The tubular, slightly curved LRD has been projected to lie horizontally, parallel to the plasma membrane [11], vertical to the plasma membrane [5], [12], [13], or at an angle to the plasma membrane [13], [14].

**Figure 1:**
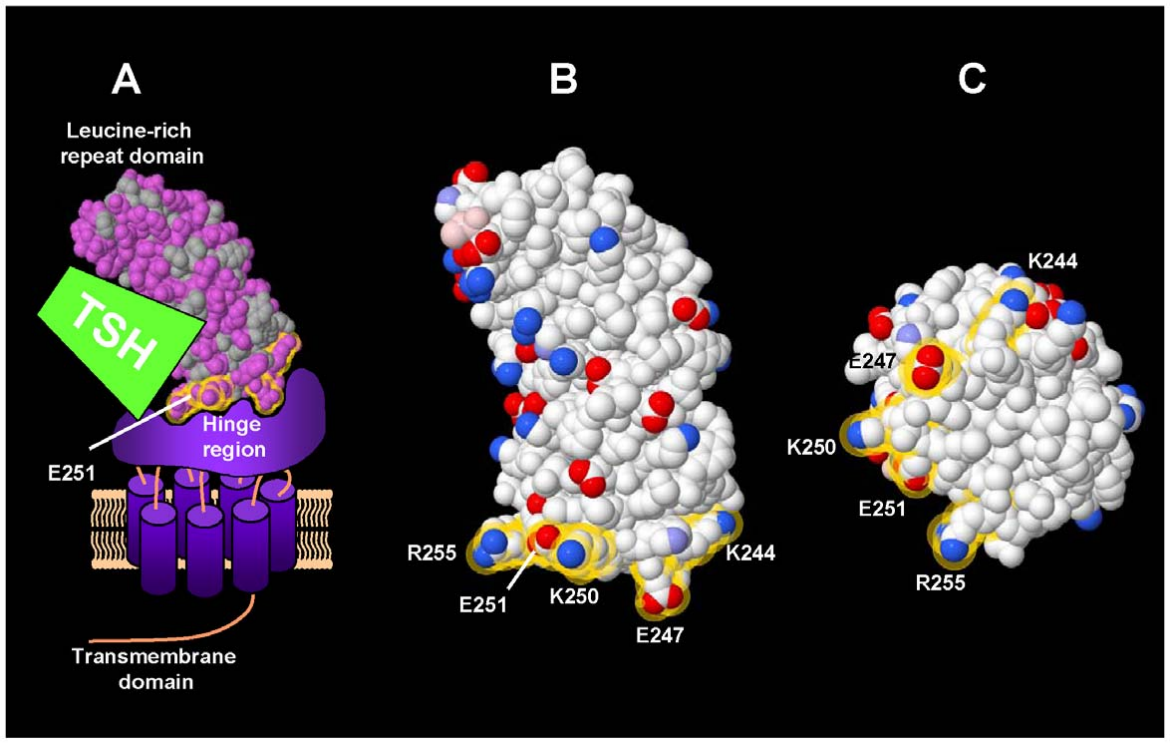
Schematic representation of the three components of the TSHR. The leucine-rich repeat domain (LRD) is linked to the serpentine transmembrane domain (TMD) by the hinge region. The crystal structure of the TSHR LRD has been solved [6](Protein Data Base 3G04) and is shown using FirstGlance in Jmol (http://molvis.sdsc.edu/fgij/). The TMD structure can be modeled with reasonable confidence from the crystal structures of other Group A, rhodopsin-like, GPCR members [1]–[4]. The structure of the intervening hinge region is totally unknown. A. Depiction of all three components. TSH binds largely to the LRD with a smaller contribution to the binding site by the hinge region. Figure 1A is a modification of Figure 6 in Ref. [15]. B. Side view of TSHR LRD. Charged residues at the C-terminal base of the LRD are indicated by the yellow halos. C. Inferior aspect of the TSHR LRD.

Recently, an inadvertent PCR cloning artifact encoding an E251K mutation in the TSHR LRD revealed reduced sensitivity to TSH stimulation despite normal high affinity TSH binding [15]. Residue E251 is situated at the base of the TSHR LRD (amino acid residues 22–260) near the junction of the LRD with the hinge region (Fig. 1A). Based on the proximity of residue E251 to the TSHR hinge region, together with the E251K mutation partially uncoupling ligand binding from signal transduction, we hypothesized that residue E251 projects into the hinge region. Uncoupling of TSH binding from TSHR signaling occurs with an E251K, but not with an E251A mutation [15]. This information suggests that E251K does not form a salt bridge with hinge residues. Rather, an E to K mutation increases the length and bulkiness of the projecting side-chain and could disrupt the normal LRD-hinge interface. Toleration of an E251A mutation is consistent with a minimal (single methyl) side-chain and stabilization by adjacent residues. The present study was based initially on the premise that amino acid residue E251 alone could not stabilize the attachment of a very large LRD to the hinge region. We, therefore, sought other TSHR LRD amino acid residues that could contribute to the stability of an LRD-hinge structural unit. These mutations yielded unanticipated information on the epitope of monoclonal antibody CS-17, a TSHR inverse agonist [16], providing insight into the structure of the receptor.

## Results

### Strategy for TSHR LRD residue mutations

Because multiple amino acids are likely to stabilize the TSHR LRD-hinge junction, we examined the TSHR LRD crystal structure for other potential residues adjacent to E251 that could interface with the hinge region and that do not contribute to the TSH binding site [17], [18]. We noted the LRD C-terminal base to be rich in additional charged amino acids, namely K244, E247, K250 and R255 (Fig. 1B and 1C). To study these residues, we chose a progressive, cumulative alanine substitution strategy. Alanine mutations would eliminate potential salt bridges without the major effect on protein conformation; as mentioned above E251A, unlike E251K, had no effect on TSH induced signal transduction [15]. By progressively ‘loosening’ the LRD-hinge interface with alanine substitutions (analogous to strands in a rope), we hypothesized that we would, at least partially, uncouple TSH binding from receptor activation.

### Effect of charged amino acid mutations at the TSHR LRD base on signal transduction

**Figure 2:**
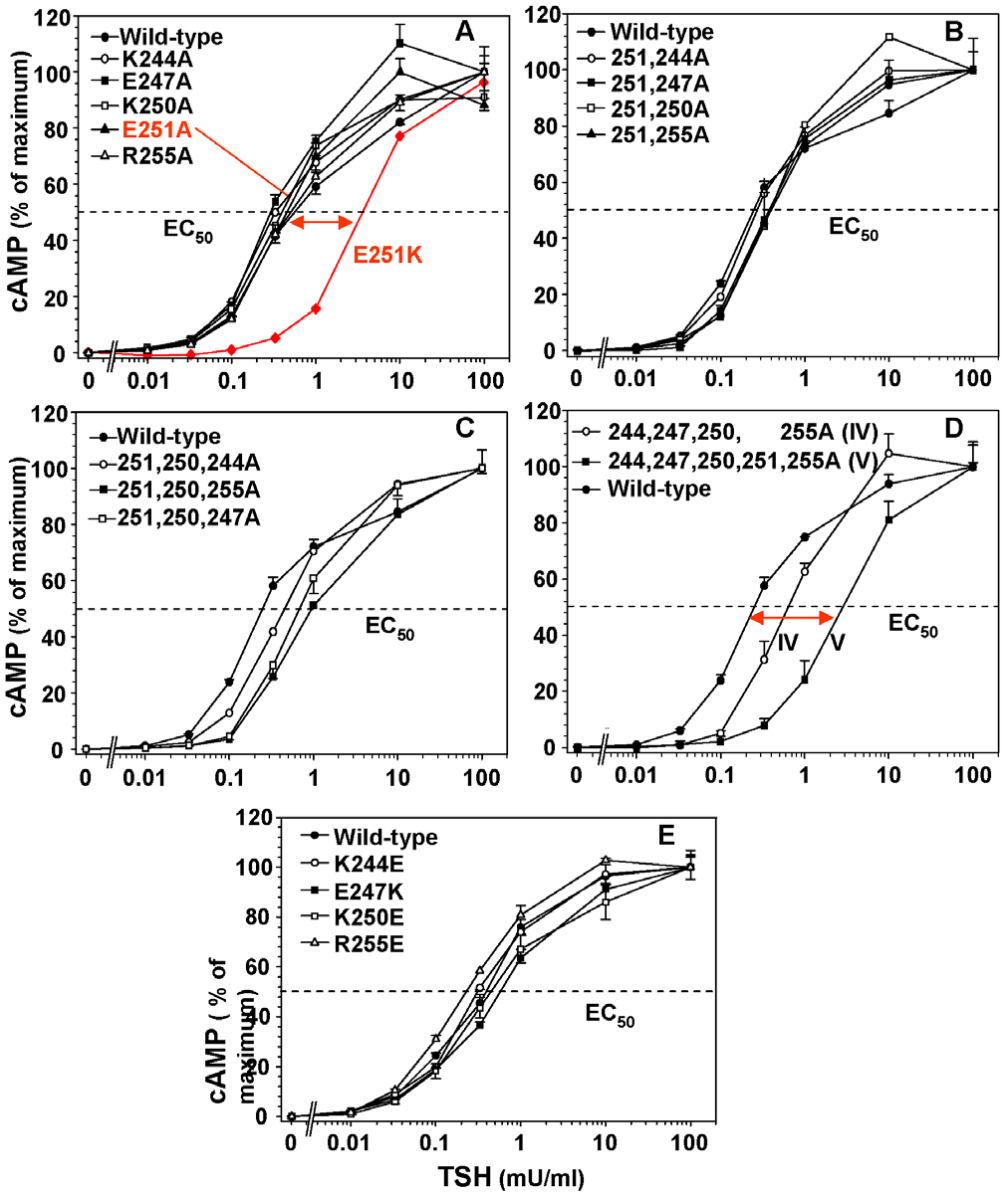
Functional effects of mutating charged residues at the C-terminal base of the TSHR LRD, singly and in combination. Unless indicated otherwise, all mutations were to alanine. CHO cells stably expressing TSHR with the indicated mutations, as well as the wild type TSHR, were incubated for 1 h in the indicated concentrations of TSH and intracellular cAMP measured (Methods). In each panel, the points represents the mean ± range of cAMP values in duplicate wells of cells. Values are expressed as a percentage of the maximal cAMP attained. The TSH effective concentrations required for half-maximal stimulation (EC_50_) in these representative experiments are indicated by the horizontal dashed line. The mean values and statistical analysis of data from multiple experiments are depicted in Figure 3. Panel A. Single mutations to alanine of charged residues at the base (C-terminus) of the TSHR LRD. For comparison, the previously published effect of an E251K mutation on the cAMP response to TSH stimulation [15] is shown in red. In the experiment shown, with basal cAMP levels of 0.8–1.3 pmoles per well of cells, mean maximum cAMP values attained were 60.5 (wild-type), 58.7 (K244A), 52.2 (E247A), 39.4 (K250A), 49.9 (E251A) and 66.1 (R255A) pmoles per well of cells. Panel B. Double mutations to alanine of charged residues at the C-terminus of the TSHR LRD. In the experiment shown, with basal cAMP levels of 0.5–0.8 pmoles per well of cells, mean maximum cAMP values were 60.5 (wild-type), 52.1 (E251,244A), 51.7 (E251A,K247A), 48.2 (E251A,K250A), and 54.7 (E251A,R255A) pmoles per well of cells. Panel C. Triple mutations to alanine of charged residues at the base of the TSHR LRD. In the experiment shown, with basal cAMP levels of 0.9–1.4 pmoles per well of cells, mean maximum cAMP values were 69.3 (wild-type), 57.9 (E251A,K250A,K244A), 55.2 (E251A,K250A,E247A) and 53.5 (E251A,K250A,R255A) pmoles per well of cells. Panel D. Combination of four (IV) and five (V) mutations to alanine of charged residues at the base of the TSHR LRD. In the experiment shown, with basal cAMP levels of 0.6–1.0 pmoles per well of cells, mean maximum cAMP values were 69.3 (wild-type), 42.9 (K244A,E247A,K250A,R255A), and 33.4 (K244A,E247A,K250A,E251A,R255A) pmoles per well of cells. Panel E. Single mutations of K244, E247, K250 and R255 to residues with an opposite charge. In the experiment shown (representative of three), with basal cAMP levels of 0.7–1.4 pmoles per well of cells, mean maximum cAMP values were 27.3 (wild-type), 31.7 (K244E), 35.8 (E247K), 34.6 (K250E) and 34.5 (R255E) pmoles per well of cells.

Compared with the wild-type TSHR, alanine substitutions for single TSHR LRD residues K244, E247, K250, E251 and R255 did not significantly alter the sensitivity of the intracellular cAMP response to TSH stimulation (effective concentrations required for 50% activation; EC50)(Fig. 2A). Similar data for E251A have been reported previously [15]. For comparison with the E251A mutation, the previously published effect of an E251K mutation on the cAMP response to TSH stimulation is also depicted (Fig. 2A, red line) [15]. The cAMP data in this and subsequent figures are shown as a percentage of the absolute cAMP attained, with the latter values provided in the figure legends.

**Figure 3:**
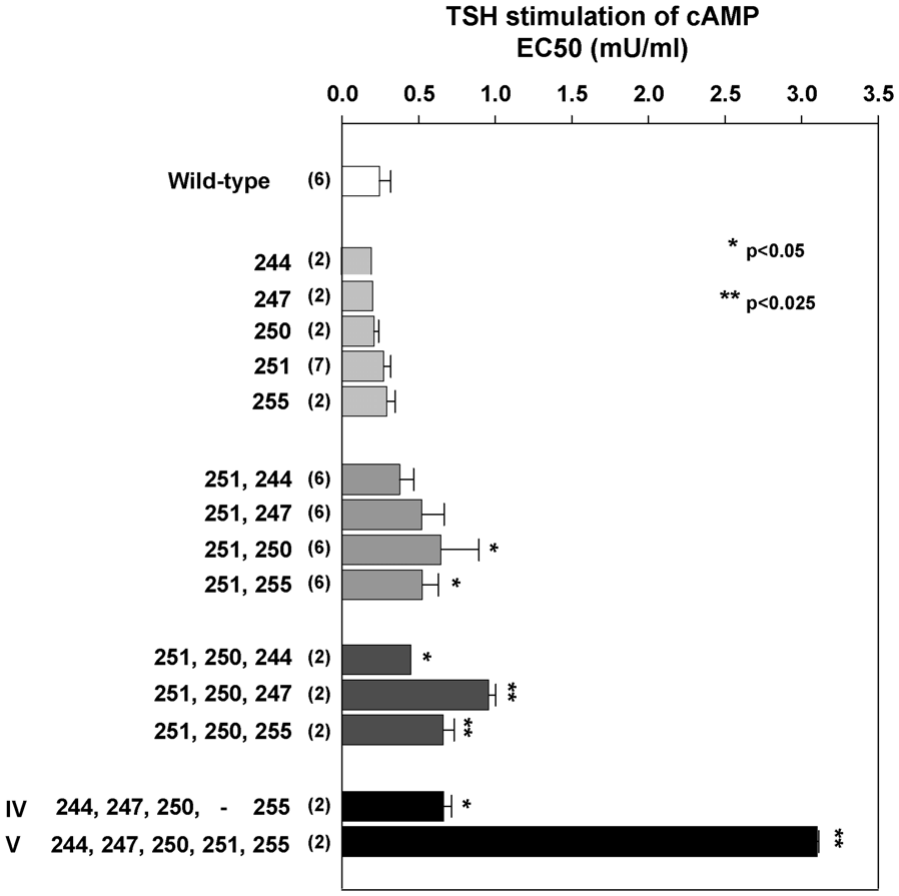
Summary and comparison of the sensitivities to TSH stimulation in all groups with alanine substitutions. The numbers besides bars indicate the amino acid residues that were mutated to alanine. Each bar represents the mean+S.D. of the EC50s for TSH stimulation of cAMP generation. The number of experiments is indicated in parentheses. Statistical comparison of each groups vs. the wild-type TSHR (wt) was performed by One-way ANOVA.

With no single amino acid mutations causing significant functional changes, we next examined dual mutations, using E251A as a template because of the known importance of the E251K mutation. The EC50 for TSH stimulation of E251,244A was similar to that of the wild-type TSHR (representative experiment shown in Fig. 2B). Small increases in the EC50 for TSH (reduced sensitivity) were observed with the three other dual mutations (E251A,K247A; E251A,K250A; and E251A,R255A)(Fig. 2B). With such small changes we studied these cell lines repeatedly. Combining the data for six experiments, the shift to the right in the EC50 with the E251A,K250A and E251A,R255A dual mutations was significant compared to the wild-type TSHR (Fig. 3). We next tested triple mutations using TSHR E251A,K250A as a template. All three mutant receptors (E251A,K250A,K244A; E251A,K250A,E247A and E251A,K250A,R255A) demonstrated small, but significant, reductions in sensitivity to TSH stimulation (Figs. 2C and 3).

When all five of the selected charged residues (K244, E247, K250, E251 and R255) were converted to alanine (for brevity termed ‘V’), the TSH EC50 was 13-fold higher than for the wild-type TSHR (3.2±0.01 vs. 0.24±0.07 mU/ml; p<0.025 ANOVA)(Figs. 2D and 3). Because E251A was common to all prior multiple mutations (double, triple and five), we examined the role of the four other residues without the E251A mutation (K244A,E247A,K250A,R255A), termed ‘IV’. This receptor, too, had reduced sensitivity to TSH stimulation, with an EC50 of 0.67±0.05 mU/ml, approximately three-fold higher than for the wild-type TSHR (p<0.05, ANOVA; Fig. 2D and 3). None of the foregoing single and multiple alanine substitutions had a significant effect on constitutive, ligand-independent TSHR activity (range of values described in the Figure legends). As with the alanine substitutions, individual mutations of the four amino acids studied to residues of the opposite charge, namely K244E, E247K, K250E and R255E, did not significantly alter the TSH EC50 for cAMP generation (Fig. 2E). In contrast, as shown in Figure 1A, an E251K mutation reduces sensitivity to TSH stimulation by approximately one order of magnitude. None of the charge inversion mutations, including E251K, led to an alteration in constitutive, ligand-independent TSHR activity (range of values in the legend to Fig. 2E).

**Figure 4:**
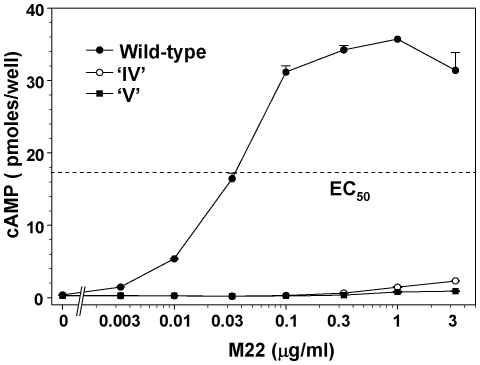
Thyroid stimulating monoclonal autoantibody M22 stimulation of TSHR variants. CHO cells stably expressing the wild-type TSHR and the TSHR with four residues (K244,E247,K250,R255) and five residues (K244,E247,K250,E251,R255) converted to alanine, termed ‘IV’ and ‘V’ respectively, were incubated for 1 h in the indicated concentrations of M22 and intracellular cAMP measured (Methods). Each point represents the mean ± range of cAMP values in duplicate wells of cells. The experiment shown is representative of three separate experiments. The very low responses of TSHR-IV and TSHR-V preclude determination of the M22 EC50's, so absolute cAMP values are shown. The M22 EC_50_ for the wild-type TSHR is indicated by the horizontal dashed line. In all other experiments in which there was a robust cAMP response, for better visualization of the EC_50_, cAMP values were normalized to 100% of the maximal value attained.

Because TSHR mutants IV and V exhibited partial uncoupling of signal transduction despite normal, high affinity TSH binding (see below), we examined whether a similar effect occurred with M22, a human monoclonal thyroid stimulating autoantibody [19]. Information from this study was potentially limited because, unlike TSH whose binding is unaffected by these receptor mutants, the crystal structure reveals R255 to be an M22 contact residue, and an R255A mutation is reported to reduce the M22 binding affinity 5-fold with a proportionate decrease in signal transduction [6]. Of note, TSHR residues K244, R247, K250 and E251 are *not* part of the M22 epitope. Relative to the wild-type TSHR, M22 had minimal activity with TSHR mutants IV and V in terms of cAMP generation (Fig. 4). The implications of this finding are discussed below (Discussion).

### TSH binding affinity to receptors with reduced sensitivity to TSH stimulation

Reduced sensitivity to TSH stimulation in TSHR mutants could be caused by a decrease in TSH binding affinity or by partial uncoupling of ligand binding and signal transduction. We focused on the TSHR with 4 and 5 alanine substitutions (IV and V) which had clearly decreased sensitivities in their functional responses to TSH. These reduced sensitivities were *not* attributable to lower TSH binding affinity. Scatchard analysis of TSH competition for ^125^I-TSH binding in four separate experiments yielded Kds for TSHR IV and V of 0.67±0.23 and 1.34±0.65 mU/ml TSH (mean ± SE), respectively, relative to a Kd of 2.40±0.30 mU/ml TSH for the wild-type TSHR studied in parallel wells of cells (Table 1). Indeed, the TSH binding affinity for TSHR IV was significantly higher than for the wild-type TSHR (p = 0.004; Student's test). However, in our experience, with varying levels of TSHR expression, such comparisons are imprecise. In particular, the relatively high level of wild-type TSHR expression can underestimate TSH binding affinity [20]. The important conclusion, however, is that independent of the wild-type TSHR, the Kd values for the TSHR IV and V mutants reflect high TSH binding affinities and, when associated with reduced sensitivity to TSH stimulation, indicate partial uncoupling between ligand binding and signal transduction.

**Table 1:** TSH binding to TSHR with four and five charged residues mutated to alanine.

		Kd(TSH mU/ml)Mean ± SE	^125^I-TSH Bound(% of total cpm)	^125^I-TSH Bound(% of wild-type)
Wild-type		2.40±0.30	29.1±1.1	100
Mutationcombination‘IV’	K244AE247AK250A(-)R255A	0.67±0.23*	16.4±3.0	55.8±9.3
Mutationcombination‘V’	K244AE247AK250AE251KR255A	1.34±0.65	11.0±1.8	37.5±5.8

TSHR IV (K244A, E247A, K250A, R255A), TSHR V (K244A, E247A, K250A, E251A, R255A) and the wild-type TSHR stably expressed in CHO cell monolayers were incubated for 4 h at room temperature in buffer containing ^125^I-TSH supplemented with the indicated concentrations of unlabeled TSH (Methods). Data from four separate experiments are expressed as the mean ± S.E.M. of radioactivity bound as a percentage of total radioactivity added per well (typically ∼30,000 cpm).

*p = 0.004, Student's t-test.

### Flow cytometric determination of TSH receptor expression level

**Figure 5:**
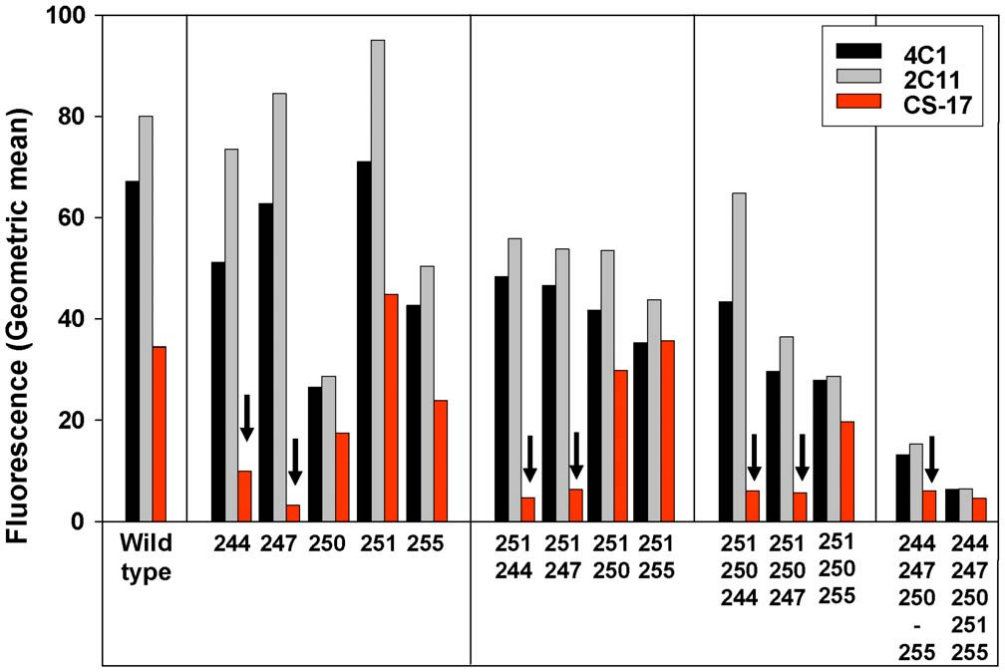
Cell surface expression by TSHR mutants determined by flow cytometry. TSHR mutants were stably expressed in CHO cells. The numbers below each bar indicate the TSHR residue mutated without, for simplicity, the specific amino acid substitution. Each cell line was tested with the three indicated monoclonal antibodies. Geometric mean fluorescence for each is net after subtraction of fluorescence obtained for each mAb with control, untransfected CHO cells (9–10 fluorescence units). Backgrounds with normal IgG controls for each mutant cell line were ∼2–6 fluorescence units. The vertical arrows indicate the low fluorescence observed with mAb CS-17 relative to the other two mAb, all such cell types involving mutation of residue K244 and/or E247.

Flow cytometry was performed on CHO cells stably expressing the wild-type and mutant TSHR using a panel of three different monoclonal antibodies (mAb) with different epitopes. The epitopes for two mAb are far removed from the LRD mutations, recognizing C-terminal portions of the hinge region close to its insertion into the plasma membrane. Thus, the epitopes for mAb 4C1 and 2C11 include residues 381–384 and 355–358, respectively [21]. In general, as measured by the latter two mAb, the level of cell surface expression progressively diminished in proportion to the number of TSHR mutations (Fig. 5). It is noteworthy that despite the low fluorescence signals for TSHR IV and V both mutants exhibited strong cAMP responses to TSH (Fig. 2D legend) and maximum ^125^I-TSH binding values (Table 1). This disparity can be explained by lesser flow cytometry sensitivity (higher threshold for detection) relative to the more sensitive functional and ligand binding procedures.

A serendipitous finding, the most important of the present study, emerged when we included mAb CS-17, a TSHR inverse agonist [16], in parallel tubes with the other two mAb. CS-17 was generated by immunization with the TSHR LRD and the N-terminal portion of the hinge region (amino acid residues 1–289). Relative to mAb 4C1 and 2C11, CS-17 recognized less well all TSHR mutants that included the K244A and E247A mutations within the LRD (Fig. 5).

### TSHR residues K244 and E247

**Figure 6:**
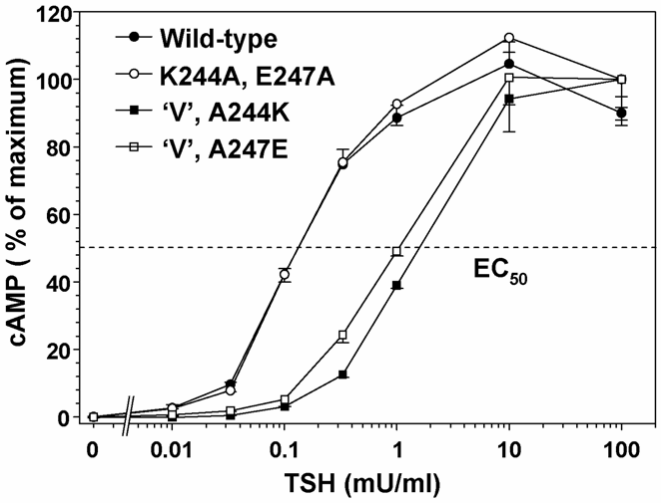
TSHR residues affecting monoclonal antibody CS-17 binding do not alter the cAMP response to TSH stimulation. CHO cells stably expressing TSHR with the indicated mutations, as well as the wild type TSHR, were incubated for 1 h in the indicated concentrations of TSH and intracellular cAMP measured (Methods). Each point represents the mean ± range of cAMP values in duplicate wells of cells. Values are expressed as a percentage of the maximal cAMP attained. The TSH effective concentration required for half-maximal stimulation (EC_50_) is indicated by the horizontal dashed line. “V” represents a receptor with mutation to alanine of five charged residues (K244,E247,K250,E251 and R255). On this background, the alanine mutations of residue 244 or 247 were reverted to the wild-type. In the representative experiment (of four) shown, with basal cAMP levels of 0.2–0.4 pmoles per well of cells, mean maximum cAMP values were 19.2 (wild-type), 20.4 (K244A, E247A), 22.8 (“V”,A244K) and 19.5 (“V”,A247E) pmoles per well of cells.

Because these two residues cannot be both water accessible (required to be part of the antibody epitope) and buried within the TSHR hinge region, such information had potentially important implications for the orientation of the LRD relative to the hinge region (see Discussion). In our earlier studies on the functional effects of alanine substitutions on the cAMP response to TSH (Fig. 2), we did not examine the combination of TSHR residues K244 and E247 independently of the other three charged residues (K250, E251 and R255). We, therefore generated a TSHR with both K244 and E247 converted to alanine. The EC50 of this mutant for TSH stimulation of cAMP generation was not different to that of the wild-type TSHR (Fig. 6). Moreover, in the receptor with all five charged residues converted to alanine (receptor “V”), whose sensitivity to TSH stimulation is reduced (Figs. 2D and 3), reversion of residues 244 and 247 back to the wild-type (A244K or A247E) did not lessen this degree of insensitivity (Fig. 6). These data indicate that TSHR residues K244 and E247 at the base of the LRD do not have a significant functional role in coupling TSH binding with signal transduction.

## Discussion

The goal of the present study was to examine the role of four charged amino acid residues adjacent to E251 at the base (C-terminus) of the TSHR LRD in coupling TSH binding and signal transduction. As is evident from Figure 1B and 1C, our choice of residues to study was limited. Other non-charged residues, probably hydrophobic, are almost certainly involved. Nevertheless, focusing on the charged residues selected for mutation, individual, dual or triple alanine substitutions of amino acid residues K244, E247, K250 and R255, as well as previously reported E251 [15], had no or a small effect on the sensitivity to TSH stimulation of intracellular cAMP generation. Yet replacement of all five residues with alanine (mutant receptor “V”) recapitulated the functional effect of the single E251K mutation. A TSHR with four alanine substitutions, leaving E251 unchanged (mutant receptor “IV) also led to partial uncoupling of TSH binding and signal transduction, but not to the same extent as in TSHR “V”. Thus, the E251A mutation (ineffective on its own) has a synergistic effect when combined with the lesser effect caused by alanine substitutions of the other four charged residues. Incidentally, although unrelated to our present study of ligand-mediated TSHR activation, ligand-independent (constitutive) TSHR activity was unaltered in the receptor mutants presented herein, as well as in receptors in which the selected charged amino acid residues were substituted with residues of the opposite charge.

The novel information provided by our present data is that, (i) E251 is dominant among the five charged residues selected for study and, (ii) when mutated as a group, adjacent amino acids, particularly K250 and R255 do, indeed, contribute to coupling TSH binding and signal transduction. These findings support the hypothesis that multiple amino acids are involved in stabilizing the TSHR LRD-hinge complex. Each alanine substitution, particularly of residue E251, loosens this attachment, analogous to progressively severing the strands on a rope. That the dual alanine substitution of K244 and E247, when added to the triple mutation of K250, E251 and R255, reduces the sensitivity to TSH stimulation despite K244 and E247 contributing to the CS-17 epitope (see below) is puzzling but could be explained by an allosteric effect on other residues at the LRD-hinge junction. Recent mutagenesis data provide further strong evidence that amino acid residues at the junction of the TSHR LRD and hinge regions further downstream to the residues studied in the present report, contribute to signal transmission following TSH binding [22].

**Figure 7:**
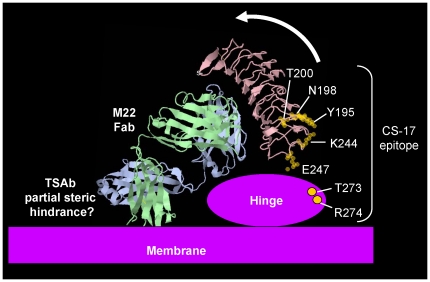
Schematic representation of the orientation of the TSHR LRD to the hinge region. The crystal structure of the TSHR LRD in complex with the M22 human monoclonal stimulating autoantibody Fab [6](Protein Data Base 3G04) is shown as a ribbon diagram using FirstGlance in Jmol (http://molvis.sdsc.edu/fgij/). For mAb CS-17 to contact TSHR residues K244 and E247 on its convex surface, these residues cannot be buried in the hinge region and the TSHR LRD is, therefore, likely to tilt forward towards its concave face, as shown by the arrow. Obviously, the M22 IgG molecule including a second Fab and an Fc is even larger than the M22 Fab and would be even more susceptible to steric hindrance, with completion of its binding by forcing out water molecules leading to torsion of the LRD vis-a-vis the hinge region. It should be noted, however, that the M22 Fab alone can activate the TSHR [19]. For simplicity, TSHR amino acid residues K250, E251 and R255 interfacing with the hinge region are omitted and are better visualized in Figure 1. Of note, in Figure 1C (inferior view of the LRD base) these three residues lie towards one pole, with CS-17 epitope residues K244 and E247 at the opposite pole. This orientation supports the concept that the LRD is tilted forward in its interface with the hinge region.

A serendipitous finding obtained with inverse agonist mAb CS-17 provides the most interesting and important information in our study. All TSHR that we investigated with either K244 or E247 replaced by alanine were recognized poorly by mAb CS-17 relative to two other antibodies with epitopes further downstream than the mutated amino acids presently studied. These data provide strong evidence that TSHR K244 and E247 contribute to the CS-17 epitope. Support for this conclusion is that from the TSHR LRD crystal structure, K244 and E247 are located on the same plane as, and very close to, other residues previously identified in the CS-17 epitope, namely Y195 [23], N198 and T200 [14](Fig. 7). Further evidence suggests that CS-17 also interacts with residues T273 and R274 in the TSHR hinge region [14], the structure of which has not been solved.

The interaction of mAb CS-17 with TSHR residues K244 and E247 indicates that the latter are water accessible and, therefore, cannot contribute significantly to the LRD-hinge interface. Based on the crystal structure of the TSHR LRD [6], for E251 (and possibly K250 and R255), but not K244 and E247, to be at the LRD-hinge interface, it is a reasonable deduction that the LRD is tilted forwards, with its concave, ligand binding surface inclined towards the hinge region and plasma membrane (Fig. 7). The present experimental data support the previous theoretical concept of the relationship between the TSHR LRD and the hinge region [13], [15].

Although the present data address the action of TSH, Figure 7 schematically depicts the binding of the monoclonal thyroid stimulating autoantibody (TSAb) Fab M22. Despite M22 and TSH both interfacing with overlapping regions on the concave surface of the TSHR LRD, the orientation of the two ligands is very different. TSH and FSH are elongated molecules that bind *transversely* to the LRD, partially wrapping around the LRD as in a hand clasp [5], [24]. M22 on the other hand binds in a more *perpendicular* manner to the LRD [6]. Further, thyroid stimulating autoantibodies [25] including M22 [26], but not TSH [27], [28], preferentially recognize the extracellular domain of the TSH holoreceptor rather than the identical extracellular domain tethered to the plasma membrane by a flexible glycosylphosphatidyl inositol (GPI) anchor. These data provide strong evidence that TSAb access to the TSHR holoreceptor is partially restricted (steric hindrance) and suggest a mechanism by which TSAb activate the receptor. Completion of TSAb binding to a partially obscured epitope could lead to torsion of the receptor transmitted via the hinge region and extracellular loops to a shift in the relative positions of the transmembrane helices. The present data suggesting that the TSHR LRD inclines forward towards its concave surface provides additional support for this concept and is also consistent with TSH making contact with both the LRD and hinge region [7]–[10].

The almost total inability of M22 to activate TSHR ‘IV’ and ‘V’ (Fig. 4) requires comment. Both receptors contain an R255A substitution, previously reported to reduce M22 binding affinity 5-fold with a proportionate reduction in cAMP generation [6]. As such, these data do not suggest uncoupling between ligand binding and signal transduction. In contrast, the near total abrogation of a functional response to M22 by TSHR-IV and -V support the concept that these combined mutations uncouple, at least in part, signal transduction for TSAb as well as for TSH. In the crystal structure of the M22-TSHR LRD complex, residue R255 is at the extreme periphery of the M22 epitope, with only part of the R255 side-chain contacting the antibody. A major portion of R255 is exposed. Because the crystal structure extends to residue T257 and the hinge is entirely absent, R255 could interface with both M22 and the hinge region. It should also be recalled that the shed A-subunit (essentially the LRD) is the likely immunogen for the generation of pathogenic TSAb in Graves' disease [25], [26], [29]. Therefore, the unrestricted access of M22 for TSHR residue R255 in the crystal structure of the isolated LRD may not occur in the TSH holoreceptor, providing further support for the concept that steric hindrance to M22 binding contributes to TSAb activation of the receptor.

Our findings also provide insight into the mechanism by which mAb CS-17 suppresses the high constitutive activity of the TSHR. Previous studies identified LRD residues Y195 [23], N198 andT200 [14], and hinge residues T273 and R274 [14] as contributing to the CS-17 epitope (Fig. 7). The present observations regarding TSHR residues K244 and E247, or the intervening loop between these two residues, support our hypothesis for the mechanism by which CS-17 exerts its inverse agonist activity [14]. Residues K244 and E247 are further downstream of residues Y195, N198 and T200, almost at the C-terminal end of the TSHR LRD (Fig. 7). Clearly, therefore, CS-17 binds to the convex dorsum of the LRD adjacent to the hinge region. These epitopic components, together with hinge residues T273 and R274 suggest that mAb CS-17 ‘fixes’ the LRD-hinge unit in a manner that reduces constitutive signaling. Because the TSHR ectodomain is itself an inverse agonist [30], [31], CS-17 may induce an LRD-hinge orientation that accentuates the intrinsic silencing of the receptor.

In summary, with respect to coupling TSH binding to TSHR activation, residue E251 is dominant among the five charged residues at the base of the LRD selected for study. However, when mutated as a group, other adjacent amino acids, particularly K250 and R255 do, indeed, contribute to coupling TSH binding and signal transduction. These data support the hypothesis that multiple amino acids are involved in stabilizing the TSHR LRD-hinge complex and are involved in transmission of a signal to the TMD. Most important, the serendipitous finding that the mAb CS-17 epitope includes LRD residues K244 and E247, near the dorsal base of the LRD, provides strong evidence that the concave surface of the LRD is tilted forward towards the membrane, providing insight into the mechanism by which ligands either activate the receptor or reduce its constitutive activity.

## Methods

### TSHR cDNA mutations

Introduction of the wild-type human TSHR (hTSHR) cDNA [32](with the H601 polymorphism converted to Y601) into the vector pcDNA5/FRT was described previously [33]. Amino acid numbering includes the signal peptide. The TSHR cDNA mutations described below in the text were generated using the QuikChange site-directed mutagenesis kit (Stratagene, San Diego, CA). All mutations were confirmed by nucleotide sequencing.

### TSHR expression

TSHR cDNAs were transfected into Flp-In-CHO cells (Invitrogen) using Fugene HD (Roche, Indianapolis IN). Cell lines stably expressing the TSHR were obtained by selection with hygromycin B (Invitrogen; ∼300 µg/ml). Cells were cultured in Ham's F12 medium supplemented with 10% fetal calf serum, penicillin (100 U/ml), gentamycin (50 µg/ml) and fungizone (2.5 µg/ml).

### Cultured cell cAMP assays

CHO cells stably expressing the wild-type or mutated TSHR were transferred into 96-well plates. For bioassay, the culture medium described above was replaced with F12 medium supplemented with 1 mM isobutyl methylxanthine (IBMX), 10 mM HEPES, 0.3% bovine serum albumin and, where indicated in the text, bovine (b) TSH (Sigma-Aldrich, St. Louis MO) or M22 [19], kindly provided by Dr. B. Rees-Smith (R.S.R. Ltd., Cardiff U.K.). Untransfected CHO cells were included as controls. After 60 min at 37 C, the medium was aspirated and intracellular cAMP was extracted with 0.2 ml 95% ethanol. The extracts were evaporated to dryness, resuspended in 0.1 ml of PBS, pH 7.5, and samples (12 µl) were assayed using the LANCE cAMP kit according to the protocol of the manufacturer (PerkinElmer, Shelton CT). The effective dose of TSH required for half maximal stimulation of intracellular cAMP levels (EC_50)_ was calculated using GraphPad Prism software (La Jolla, CA).

### TSH binding

CHO cells expressing the TSHR were cultured in 24-well plates. Medium was aspirated and replaced with 250 µl binding buffer (Hanks' buffer with 250 mM sucrose substituting for NaCl to maintain isotonicity and 0.25% bovine serum albumin) containing ∼30,000 cpm ^125^I-TSH (B.R.A.H.M.S, Berlin Germany) and the indicated concentrations of unlabeled bTSH. After incubation for 4 h at room temperature, cells were rapidly rinsed three times with binding buffer (4°C), solubilized with 0.2 ml 1 N NaOH, and radioactivity was then measured in a **γ**-counter. TSH binding affinities were measured by Scatchard analysis using GraphPad Prism (La Jolla, CA), excluding the low affinity, high capacity non-specific binding site to which TSH (unlike the other glycoprotein hormone receptors) is susceptible. Non-linear regression analysis does not provide reliable information in all TSHR binding experiments.

### Flow Cytometry

CHO cells were harvested from 6 well plates using 1 mM EDTA, 1 mM EGTA in PBS. After washing twice with PBS containing 10 mM HEPES, pH 7.4, 2% fetal bovine serum, and 0.05% NaN_3_, the cells were incubated for 30 min at room temperature in 100 µl of the same buffer containing 1 µg of either normal mouse IgG or murine mAb 2C11 and 4C1 (Morphosys, Raleigh NC), as well as mAb CS-17 [16]. After rinsing, the cells were incubated for 45 min with 100 µl fluorescein isothiocyanate-conjugated goat anti-mouse IgG (1∶100) (Caltag, Burlingame, CA), washed, and analyzed using a Beckman FACScan flow cytofluorimeter. Cells stained with propidium iodide (1 µg/ml final concentration) were excluded from analysis.
